# Bepridil monotherapy failed to prevent coronary vasospasm in a Brugada syndrome patient

**DOI:** 10.1093/omcr/omac082

**Published:** 2022-08-18

**Authors:** Takuro Kazatani, Akinori Higaki, Yuta Tanaka, Yoshitaka Kawada, Go Hiasa, Tadakatsu Yamada, Hideki Okayama

**Affiliations:** Department of Cardiology, Ehime Prefectural Central Hospital, Matsuyama, Japan; Department of Cardiology, Ehime Prefectural Central Hospital, Matsuyama, Japan; Department of Cardiology, Pulmonology, Hypertension & Nephrology, Ehime University Graduate School of Medicine, Toon, Japan; Department of Cardiology, Ehime Prefectural Central Hospital, Matsuyama, Japan; Department of Cardiology, Ehime Prefectural Central Hospital, Matsuyama, Japan; Department of Cardiology, Ehime Prefectural Central Hospital, Matsuyama, Japan; Department of Cardiology, Ehime Prefectural Central Hospital, Matsuyama, Japan; Department of Cardiology, Ehime Prefectural Central Hospital, Matsuyama, Japan

## Abstract

Coronary vasospasm sometimes coexists with Brugada syndrome (BrS) and is reportedly associated with poor prognosis. Although calcium channel blockers are considered first-line drugs to prevent coronary vasospasm, they also have the potential to induce ST elevation and ventricular fibrillation (VF) in BrS. Therefore, the optimal medication for such a complicated case is still underdetermined. We report a male patient who presented with VF due to BrS, which was later found to have coexisted with coronary vasospasm. He was treated with low-dose bepridil expecting both its anti-arrhythmic and vasodilatory effects, but a later acetylcholine provocation test showed no suppression of vasospasm. Based on these results, we decided to add nitrates to the medication. This case report illustrates that drug selection needs caution in BrS when complicated with vasospastic angina and that bepridil monotherapy may not be sufficient to suppress coronary vasospasm in such cases.

## INTRODUCTION

Brugada syndrome (BrS) is one of the important causes of sudden cardiac death in young people [1]. It is known that 11–13% of BrS patients have intercurrent coronary vasospasm [2]. It has also been reported that coronary artery vasospasm is associated with cardiac events in BrS patients with an implantable cardiac defibrillator (ICD) [3]. Although calcium channel blockers (CCBs) are considered first-line drugs to prevent coronary vasospasm, they also have the potential to induce ST elevation and ventricular fibrillation (VF) in BrS [4–6]. Therefore, effective medical treatment for BrS with coronary vasospasm is still under debate [7].

## CASE REPORT

**Figure 1:**
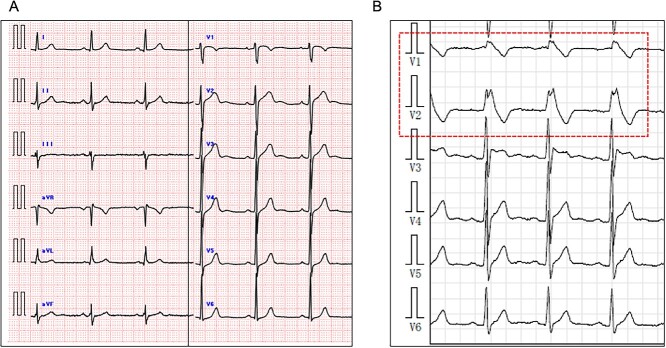
ECGs at different time points. Panel **A** shows a 12-lead ECG after the completion of therapeutic hypothermia. Panel **B** shows the Holter ECG during the pilsicainide administration test. A type 1 ST-segment elevation was observed in V1 and V2 (red dashed line frame).

**Figure 2:**
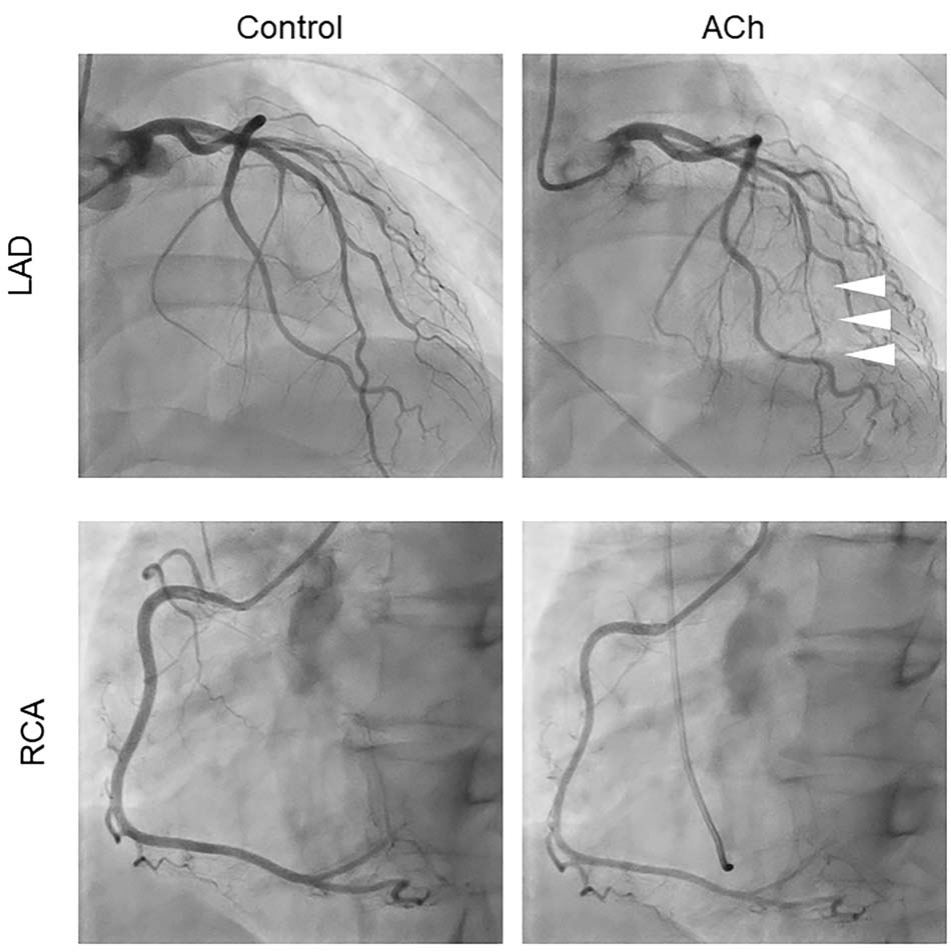
The initial acetylcholine provocation test. No spasm occurred in the right coronary artery (RCA), but sub-occlusion was observed in the left anterior descending artery (LAD) after administration of 50 μg and 100 μg of acetylcholine (arrowheads).

**Figure 3:**
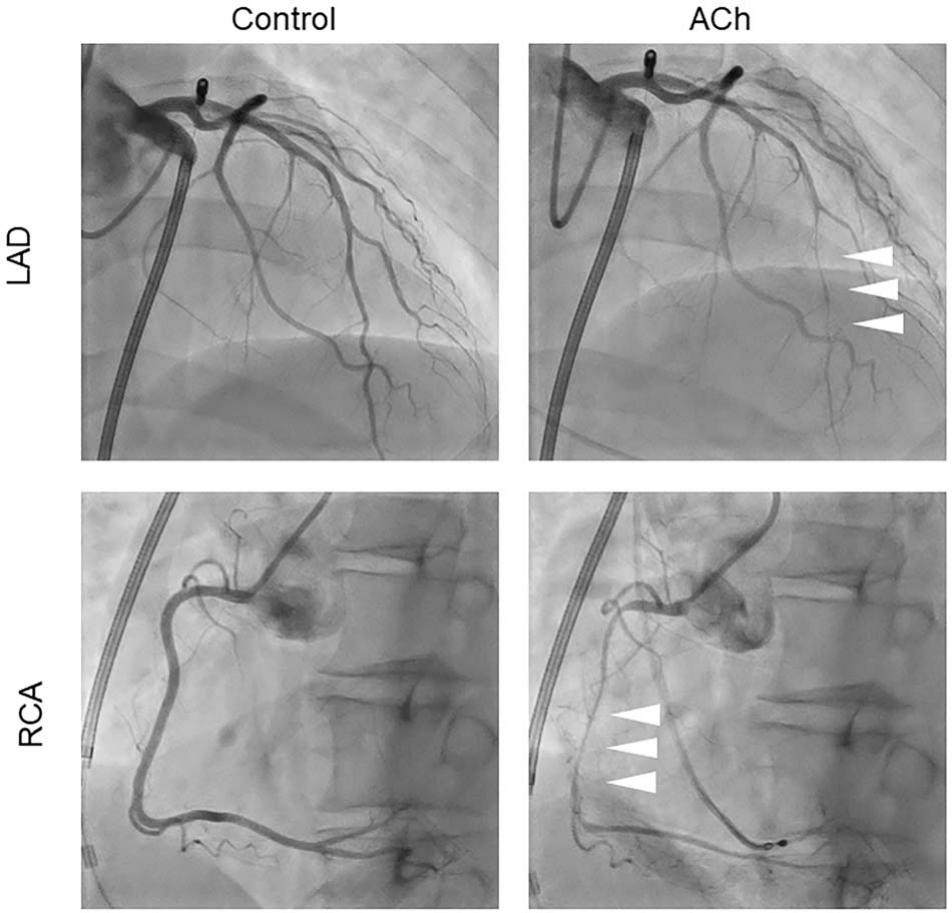
Acetylcholine provocation test under treatment with bepridil. Severe stenosis and sub-occlusion were observed in the right coronary artery (RCA) and left anterior descending artery (LAD) after administration of 50 μg of acetylcholine (arrowheads).

The case is a 46-year-old male with a smoking history. Although he was pointed out to have a Brugada-type electrocardiogram (ECG) in a past occupational medical health checkup, he was observed without further examination because of a lack of symptoms and family history. One day in the early morning, he lost consciousness at home, and an ambulance was called by his family. Upon the arrival of the emergency services, he was in cardiac arrest due to VF and was taken to our hospital after successful resuscitation for a VF with an automatic external defibrillator. In the emergency department, his ECG showed idioventricular rhythm with a heart rate of 110/min, and no obvious ST elevation was observed. The echocardiography showed a left ventricular ejection fraction of 60% with normal wall motion and valvular function. The emergency coronary angiography showed no significant stenosis, and he was tentatively diagnosed with idiopathic VF and was admitted to the intensive care unit. He underwent target temperature management (35°C for 24 h) and regained consciousness 12 h after the rewarming without neurological deficit. The follow-up 12-lead ECG showed early repolarization, but a typical Brugada-type ECG was not observed (Fig. 1**A**). Considering the possibility of VF due to coronary vasospasm, we performed an acetylcholine provocation test on the fifth hospital day. No spasm occurred in the right coronary artery, but sub-occlusion was observed in the left anterior descending artery after administration of 50 μg and 100 μg of acetylcholine (Fig. 2). On the other hand, he did not complain of chest symptoms and no significant changes in the ECG were observed during the spasm provocation. On the ninth hospital day, a pilsicainide administration test was performed. Immediately after the drug administration, ST-segment elevation with the right bundle branch block was observed in V1 and V2 (Fig. 1**B**). Based on these findings, he was diagnosed as VF due to BrS. He was implanted with a subcutaneous ICD on the 12th hospital day to prevent sudden cardiac death. He started taking bepridil at 100 mg/day to suppress VF recurrence and was discharged on the 16th hospital day. A month after discharge, an acetylcholine provocation test was performed once again to evaluate the efficacy of bepridil on the coronary vasospasm. As a result, the coronary spasm was induced by 50 μg of acetylcholine in both the right coronary artery and the left coronary artery (Fig. 3). Based on this result, we decided to add nitrates to the medication. His subsequent course was uneventful and being followed up at the outpatient clinic on a regular basis.

## DISCUSSION

Drugs that have been reported to prevent VF in BrS include quinidine, cilostazol and bepridil [8]. Although bepridil is classified as CCB, it can also suppress multiple K channels including Ito, and the subsequent upregulation of Na channels is thought to suppress VF via increasing Na current [9]. Although CCB is the first choice for the treatment of vasospastic angina, there is a concern that suppression of inward Ca current in BrS may increase ventricular arrhythmia [4, 5]. It has also been reported that K-channel opening drugs, known as vasodilators, induced coved-type ST elevation [10]. In addition to the above-mentioned antiarrhythmic effect, bepridil has been reported to be safe and effective in stable angina [11, 12]. Therefore, we expected this drug to have an inhibitory effect on both coronary spasm and arrhythmia [13]. However, in our case, the coronary spasm was not suppressed by bepridil monotherapy, but rather worsened in the second acetylcholine provocation test. Therefore, we decided to add nitrate to suppress coronary spasms, in accordance with precedent [5]. Since nitrate tolerance can develop with long-term use, and the possible calcium channel blocking effects of nitrates have been pointed out [14, 15], their efficacy should be evaluated periodically.

Brugada phenocopies are clinical entities in which the diagnostic criteria for BrS are not fulfilled, but the Brugada-type ECG is induced by certain clinical conditions [16]. Previously, Itoh *et al*. reported a case of vasospastic angina presenting Brugada-type ECG abnormalities [17]. In their case, combination therapy with diltiazem and flecainide could suppress both anginal symptoms and syncope caused by VF. However, our case differs in that acetylcholine administration did not induce Brugada-type ECG or VF, nor did it provoke any chest symptoms.

One might surmise that the coronary spasm could not be suppressed due to the low dose of bepridil, but there was a concern about the dose-dependent QT-prolonging effect. Since it has been reported that low-dose bepridil is effective enough for BrS with SCN5A mutation [18], we adopted 100 mg/day in this case. As drug options are limited in BrS with coronary spasms, further research is needed to clarify the true effects of bepridil.

## CONFLICTS OF INTEREST STATEMENT

None declared.

## ETHICAL APPROVAL

The case report was approved by the Ethical Committee of Ehime Prefectural Central Hospital.

## CONSENT

Informed consent was obtained from the patient.
